# Correlation of systemic immune inflammation index and systemic inflammatory response index with the severity of Parkinson’s disease

**DOI:** 10.3389/fneur.2026.1736318

**Published:** 2026-01-23

**Authors:** Fangyi Li, Zhen Wang, Mingzhu Deng, Jian Peng, Guohua He, Yangping Tong, Wei Xu, Tieqiao Feng, Kangping Song

**Affiliations:** 1 Department of Neurology, The Affiliated Changsha Central Hospital, Hengyang Medical School, University of South China, Changsha, Hunan, China; 2 Department of Neurology, The Second People’s Hospital of Hunan Province (Brain Hospital of Hunan Province), Changsha, Hunan, China

**Keywords:** associated factor, inflammation, Parkinson’s disease, systemic immune inflammation index, systemic inflammatory response index

## Abstract

**Background:**

While the significance of inflammation in Parkinson’s disease (PD) pathogenesis has been established, the relevance of emerging hematological markers such as the systemic immune-inflammation index (SII) and systemic inflammatory response index (SIRI) to this disorder requires further investigation.

**Methods:**

Whole blood were collected and analysed for the measured parameters from 222 Parkinson’s disease (PD) patients and 298 healthy controls (HCs), All PD patients undergoing comprehensive neuropsychological assessment. Partial correlation analysis was used to evaluate the correlation between SII, SIRI and PD severity, after adjusting for age. Logistic regression models were constructed to evaluate the associations of these inflammatory indices with PD risk, while receiver operating characteristic (ROC) analysis assessed their diagnostic performance.

**Results:**

The SII and SIRI were substantially higher in patients with PD than in HCs. Both the SII and SIRI were positively correlated with Hoehn and Yahr staging scale (H&Y), Unified Parkinson’s Disease Rating Scale (UPDRS), UPDRS-I, UPDRS-II, and UPDRS-III scores. Conversely, the SII exhibited a negative relationship with Mini-Mental State Examination (MMSE) scores. A binary logistic regression model demonstrated that the SII [odds ratio (OR), 1.601; 95% confidence interval (CI) 1.484–1.828, *p* < 0.001] and SIRI (OR, 1.487; 95% CI, 1.319–1.609, *p* < 0.001) were independent factors for PD. The area under the curve (AUC) values for the SII, SIRI, and SII & SIRI for PD were 0.750, 0.700, and 0.785, respectively.

**Conclusion:**

Our findings support the potential utility of elevated SII and SIRI as biomarkers for assessing PD severity.

## Introduction

Parkinson’s disease (PD) is a chronic, progressive neurodegenerative disorder predominantly characterized by motor dysfunction (1, 2). The core pathological features include degeneration of dopaminergic neurons in the substantia nigra and the presence of Lewy bodies (3). Globally, the number of individuals with PD is projected to exceed 14 million by 2040, a trend particularly pronounced in developing countries (4). To date, no therapy exists to cure PD or halt its progression. The disease substantially impairs patients’ quality of life and reduces life expectancy, while also imposing a considerable socioeconomic burden. Although biomarkers from cerebrospinal fluid, blood, and peripheral tissues show promise for early detection, and advanced neuroimaging techniques, including positron emission tomography (PET), single-photon emission computed tomography (SPECT),and magnetic resonance imaging (MRI), enable detailed visualization of neurodegeneration and related cerebral changes (5), the widespread clinical adoption of these approaches remains constrained by high costs and invasive sampling requirements.

Neuroinflammation represents a well-established pathological component of PD (6–8). Chronic microglial activation contributes substantially to neuronal loss through the release of proinflammatory cytokines and reactive oxygen species, which subsequently disrupt blood–brain barrier (BBB) integrity and facilitate peripheral immune cell infiltration into the central nervous system. This cascade ultimately amplifies neurodegenerative processes (9). Peripheral inflammatory alterations in PD further involve neutrophil hyperactivation and enhanced lymphocyte migration across the compromised BBB (1, 10, 11). Recent attention has focused on composite inflammatory indices, particularly the systemic immune-inflammation index (SII) and systemic inflammatory response index (SIRI) (12). By integrating neutrophil, platelet, and lymphocyte counts, SII provides a comprehensive assessment of immune-inflammatory balance. Elevated SII values have been correlated with unfavorable outcomes across various conditions, including solid tumors, thromboembolic events, cardiovascular diseases, and COVID-19 (13–16). Although emerging evidence highlights the potential significance of SII, its association with PD remains incompletely characterized. While one study identified serum SII as a determinant of motor performance in PD patients (17), and recent studies revealed a positive correlation between SII and PD incidence in U.S. adults (18, 19). However, the association between SII and PD remains contentious. Two studies reported no significant association between elevated SII levels and PD risk (20, 21). Thus, the role of SII in PD warrants further investigation. Similarly, SIRI has emerged as a novel marker of systemic inflammatory status, demonstrating prognostic utility in pneumonia, rheumatoid arthritis, acute pancreatitis (22–24), and cardiovascular events (25–28). Although one study documented a positive correlation between SIRI and anxiety symptoms in PD patients (29), the overall relationship between SIRI and PD remains undefined.

Given the established role of neuroinflammation in PD. Since the associations of the SII and SIRI with PD remain unclear, this study was designed to evaluate their potential clinical relevance as progression indicators.

## Materials and methods

### Study design and participants

**Figure 1:**
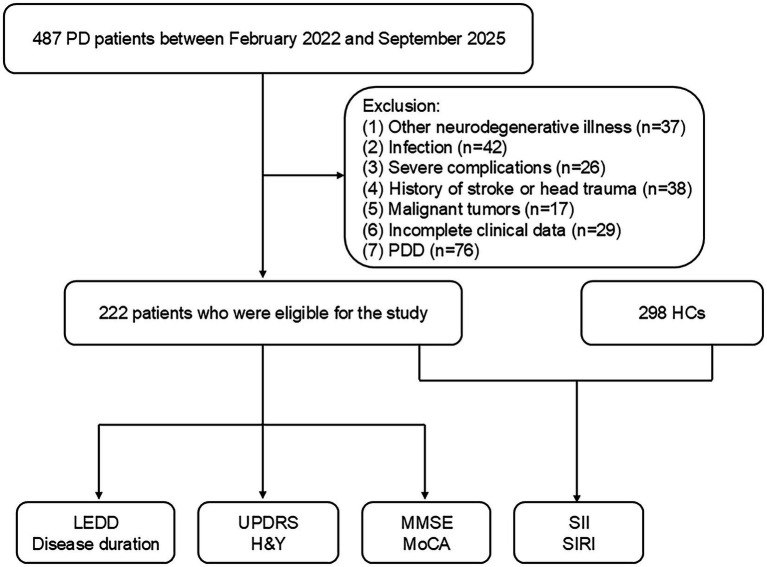
Flow diagram of participant screening and enrollment. SIRI, systemic inflammation response index; SII, systemic inflammation index; MMSE, Mini-Mental State Examination; MoCA, Montreal Cognitive Assessment; H&Y, Hoehn and Yahr staging scale; UPDRS, Unified Parkinson’s Disease Rating Scale; LEDD, levodopa equivalent daily dose; HCs, healthy controls; PD, Parkinson’s disease, PDD, Parkinson’s disease dementia.

PD patients were recruited from The Second People’s Hospital of Hunan Province and Changsha Central Hospital. The study protocol received ethical approval from both institutional review boards, and all participants provided written informed consent. PD diagnosis followed the International Parkinson and Movement Disorder Society criteria (30). Inclusion required complete clinical and laboratory data. Exclusion criteria encompassed: (1) other neurodegenerative disorders (e.g., Alzheimer’s disease, frontotemporal dementia); (2) hematological/immunological diseases, malignancy, hypothyroidism, or active infection; (3) severe hepatic/renal dysfunction; (4) history of cerebrovascular events or traumatic brain injury; (5) Parkinson’s disease dementia; and (6) hypertension, cardiovascular and cerebrovascular disease, diabetes, and psychiatric disorders. Healthy controls were enrolled from health examination centers at both hospitals using identical exclusion criteria. Ultimately, 222 PD patients were enrolled between February 2022 and September 2025 (Figure 1).

### Blood collection and clinical evaluation

Trained neurologists, blinded to participant grouping, conducted all clinical assessments. We recorded baseline characteristics (age, sex, height, weight, education) and calculated body mass index (BMI) as weight in kilograms divided by height in meters squared (kg/m^2^). Each PD patient underwent comprehensive evaluation using the following standardized instruments: levodopa equivalent daily dose (LEDD), the Unified Parkinson’s Disease Rating Scale (UPDRS) with its non-motor (UPDRS-I), activities of daily living (UPDRS-II), and motor (UPDRS-III) subscales, and the Hoehn and Yahr (H&Y) stage for disease progression. Cognitive function was evaluated comprehensively using both the Montreal Cognitive Assessment (MoCA) and the Mini-Mental State Examination (MMSE).

Fasting venous blood was collected from all participants between 6:00 and 7:00 a.m. after an 8-h overnight fast. Complete blood count analysis was performed on EDTA-anticoagulated samples using an automated hematology analyzer (BZ6800, China), while biochemical parameters were measured from clotted blood using an automated analyzer (HITACHI 7600, Japan). All assays employed commercial kits and were conducted in triplicate by trained personnel following standardized protocols. Measured parameters included white blood cells (WBC), neutrophils (N), lymphocytes (L), monocytes (M), platelets (P), creatinine (Cr), and uric acid (UA). The systemic immune-inflammation index (SII) and systemic inflammation response index (SIRI) were calculated as SII = P × (N/L) and SIRI = N × (M/L), respectively (12).

### Statistical analyses

Statistical analyses were conducted using SPSS 25.0 (IBM Corp., Armonk, NY, USA). The normality of continuous variables was assessed with the Kolmogorov–Smirnov test. Normally distributed data are presented as mean ± standard deviation (SD), and nonnormally distributed data as median with interquartile range (IQR). Categorical variables are summarized as frequencies (percentages). Group comparisons were performed using the Student’s t-test (normal distribution) or the Mann–Whitney U/Kruskal–Wallis test (nonnormal distribution). Correlations were examined with Pearson or Spearman methods based on data distribution. Partial correlation analysis between clinical characteristics and inflammatory parameters in PD patients, after adjusting for age. Multicollinearity was assessed by calculating the variance inflation factor (VIF) for all independent variables. Multicollinearity among independent variables was evaluated before constructing a binary logistic regression model to identify PD associated factor. The diagnostic performance of inflammatory biomarkers was evaluated using receiver operating characteristic (ROC) curve analysis, with a two-tailed *p* < 0.05 considered statistically significant.

## Results

### Comparison of baseline clinical and demographic profiles between patients with PD and HCs

**Figure 2:**
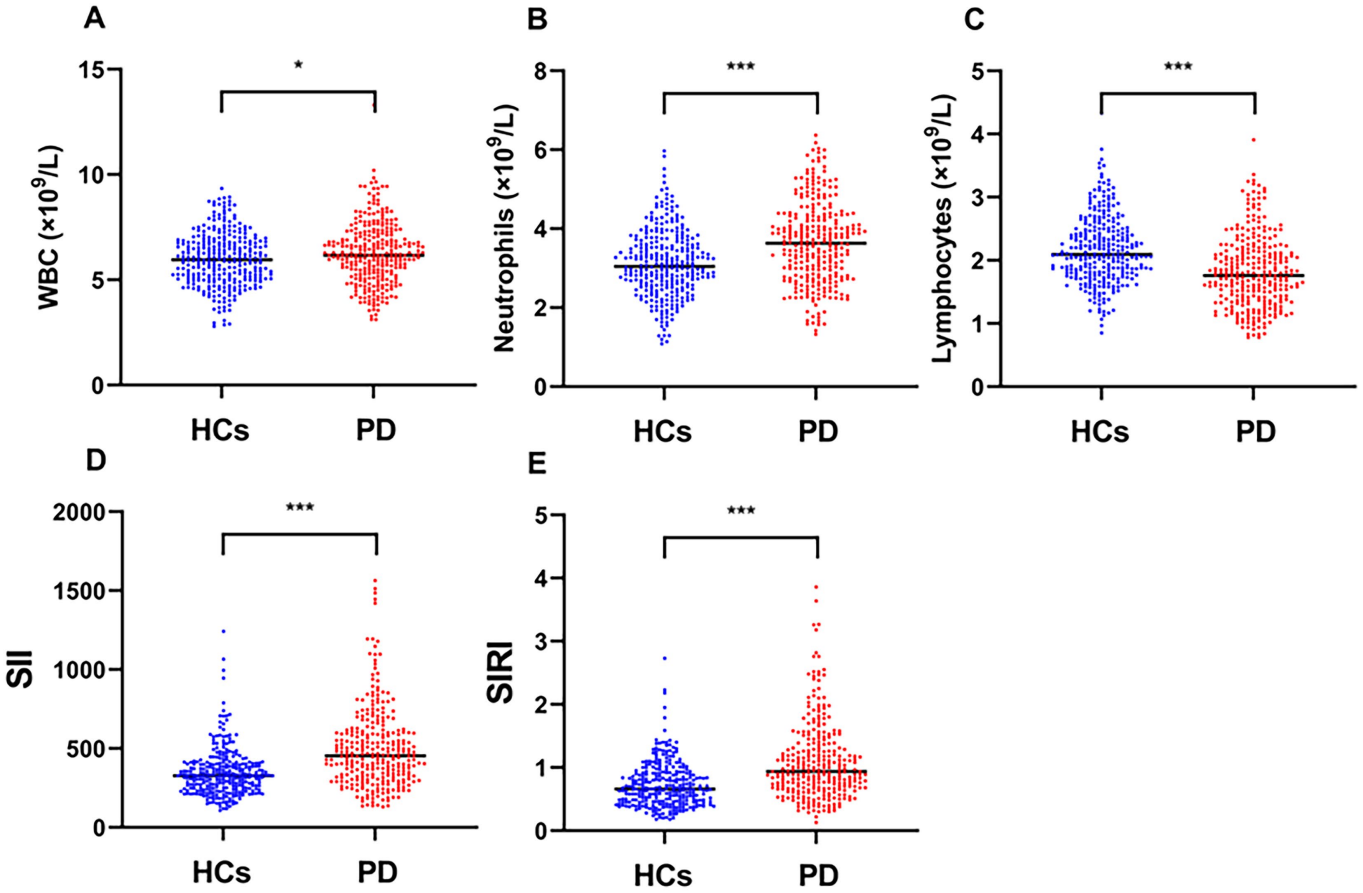
Comparison of **(A)** WBC, **(B)** neutrophil, **(C)** lymphocyte, **(D)** SII, and **(E)** SIRI between patients with PD and HCs. *p < 0.05, **p < 0.01, ***p < 0.001.

Table 1 summarizes the baseline demographic and clinical characteristics of the study cohort, which comprised 222 PD patients (53.60% male) and 298 HCs (49.66% male). Among PD patients, 59.01% (*n* = 131) presented with the AR/PIGD subtype, 12.61% (*n* = 28) with the TD subtype, and 28.38% (*n* = 63) with a mixed subtype at disease onset. The PD group showed significantly higher WBC (*p* = 0.013), neutrophils (*p* < 0.001), SII (*p* < 0.001), and SIRI (*p* < 0.001) than the HCs group, but significantly lower lymphocytes (*p* < 0.001) than the HCs group. In addition, Figure 2 shows the WBC, neutrophils, lymphocytes, SII and SIRI for the two groups.

**Table 1:** Clinical and demographic characteristics of patients with PD and HCs.

Variable	PD patients (*n* = 222)	HCs (*n* = 298)	*Z*/*T*	*p*
Male, *n* (%)	119 (53.60)	148(49.66)	0.790	0.374
Age, years	65.58 ± 9.28	63.79 ± 8.78	−1.924	0.093
BMI, kg/m^2^	23.04 ± 3.15	24.65 ± 3.12	−1.151	0.113
Education	9 (6–12)	9(7–13)	−2.221	0.142
Duration, years	4 (2–7)			
AR/PIGD subtype, *n* (%)	131 (59.01)			
TD subtype, *n* (%)	28 (12.61)			
Mixed subtype, *n* (%)	63 (28.38)			
UPDRS	39 (26.38–52)			
UPDRS (I)	3 (2–5)			
UPDRS (II)	12 (8–15)			
UPDRS (III)	22 (15–32)			
H&Y	2.5 (2–3)			
MMSE	27 (24–29)			
MoCA	27 (23–28)			
LEDD, mg	533.62 (374–702.32)			
WBC (×10^9^/L)	6.24 ± 1.48	5.98 ± 1.27	−1.981	0.013
Platelets (×10^9^/L)	222.50 (191.00–259.98)	226 (191.75–267.25)	−0.316	0.752
Neutrophils (×10^9^/L)	3.71 (2.72–4.39)	3.05 (2.56–3.64)	−5.642	<0.001
Monocytes (×109/L)	0.47 (0.38–0.59)	0.46 (0.38–0.57)	−1.054	0.292
Lymphocytes (×10^9^/L)	1.77 (1.41–2.15)	2.10 (1.80–2.49)	−7.827	<0.001
Cr (μmol/L)	71.30 (61.23–85.02)	69(59–80.48)	−1.720	0.086
UA (μmol/L)	323.63 ± 92.87	334.81 ± 77.73	1.491	0.136
SII	455.83 (320.19–605.23)	327.89 (246.50–402.96)	−8.502	<0.001
SIRI	0.96 (0.64–1.38)	0.66 (0.46–0.91)	−7.849	<0.001

BMI, body mass index; WBC, white blood cells; R/PIGD, akinetic-rigid/postural instability gait difficulty; TD, tremor-dominant; Cr, creatinine; UA, uric acid; H&Y, Hoehn & Yahr; PDD, Parkinson’s disease dementia.

### Partial correlation analysis between clinical characteristics (UPDRS, H&Y, MoCA, MMSE, disease duration, and LEDD) and inflammatory parameters (SII, SIRI, neutrophils, lymphocytes, WBC) in PD patients

Due to immunosenescence process, we performed partial correlation analyses with adjustment for age. The SII showed significant positive correlations with H&Y (*r* = 0.486, *p* < 0.001), total UPDRS score (*r* = 0.458, *p* < 0.001), and its subscales (UPDRS-I: *r* = 0.455; UPDRS-II: *r* = 0.403; UPDRS-III: *r* = 0.455; all p < 0.001) (Table 2). The SIRI was positively correlated with H&Y (*r* = 0.408, *p* < 0.001), total UPDRS score (*r* = 0.411, *p* < 0.001), and all UPDRS subscales (UPDRS-I: *r* = 0.302; UPDRS-II: *r* = 0.307; UPDRS-III: *r* = 0.385; all *p* < 0.001) (Table 2).

**Table 2:** Partial correlation analysis of clinical parameters in patients with PD.

Variable	SII		SIRI		WBC		Neutrophils		Lymphocytes	
	*r*	*p*	*r*	*p*	*r*	*p*	*r*	*p*	*r*	*p*
H&Y	0.486	<0.001	0.408	<0.001	0.059	0.152	0.162	0.321	−0.178	0.113
UPDRS	0.458	<0.001	0.411	<0.001	0.056	0.311	0.133	0.113	−0.067	0.733
(I)	0.455	<0.001	0.302	<0.001	0.111	0.095	0.150	0.099	−0.032	0.427
(II)	0.403	<0.001	0.307	<0.001	0.104	0.065	0.131	0.112	−0.038	0.602
(III)	0.455	<0.001	0.385	<0.001	0.018	0.763	0.044	0.452	−0.119	0.112
MMSE	−0.206	0.113	−0.102	0.095	−0.052	0.363	−0.015	0.194	0.105	0.136
MOCA	−0.097	0.107	−0.064	0.362	−0.006	0.911	−0.061	0.223	0.097	0.114
Duration	0.142	0.053	0.170	0.071	0.030	0.963	0.106	0.116	−0.186	0.105
LEDD	0.093	0.166	0.107	0.112	0.055	0.416	0.103	0.126	−0.063	0.353

### Logistic regression analysis of associated factor for PD

To identify independent associated factor for PD, we performed binary logistic regression incorporating all variables showing significance in univariate analyses (Table 1). In addition, sex, age, UA level, and BMI—all previously reported to be associated with Parkinson’s disease—were included as covariates in the multivariate regression models. The results of these models are presented in Table 3. Variance inflation factors (VIF) indicated no collinearity between SII and SIRI (VIF = 1.41), though neutrophils (VIF = 40) and lymphocytes (VIF = 37) were excluded due to high collinearity. After controlling for male, age, BMI and WBC, the SII (OR, 1.601; 95% CI 1.484–1.828, *p* < 0.001), SIRI (OR, 1.487; 95% CI 1.319–1.609, p < 0.001) were found to be biomarkers for assessing PD severity for PD (Table 3).

**Table 3:** Logistic regression analysis for associated factor for PD.

Variable	OR (95% CI)	*p*	Adjusted OR (95% CI)	*p*
Male	1.143 (1.092–1.349)	0.162	1.103 (1.083–1.211)	0.121
Age	1.115 (1.009–1.216)	0.077	1.098 (1.041–1.189)	0.185
BMI	0.810 (0.781–0.991)	0.131	0.934 (0.809–0.997)	0.241
WBC	1.151 (1.024–1.300)	0.036	1.096 (1.029–1.271)	0.147
Neutrophils	1.748 (1.437–2.031)	<0.001		
Lymphocytes	0.819 (0.631–0.984)	0.007		
UA	0.899 (0.675–0.992)	0.101	0.924 (0.788–0.982)	0.124
SII	1.847 (1.632–2.041)	<0.001	1.601 (1.484–1.828)	<0.001
SIRI	1.608 (1.601–1.826)	<0.001	1.487 (1.319–1.609)	<0.001

### Subgroup analyses and interaction test

**Figure 3:**
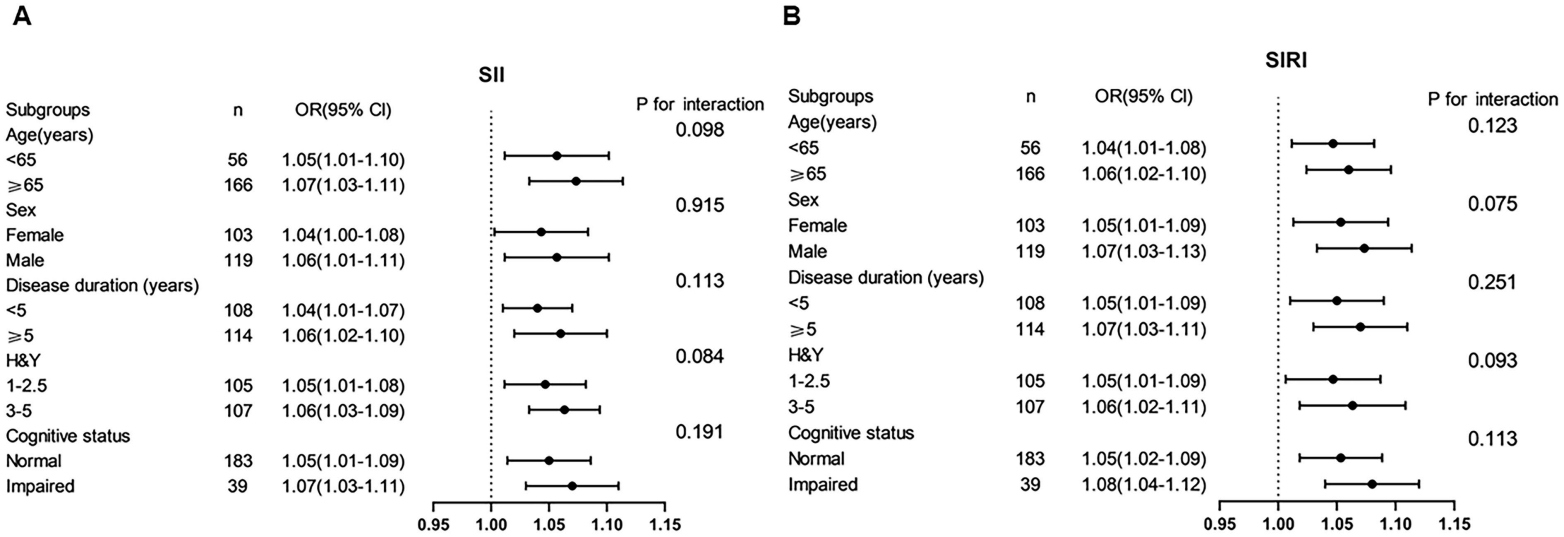
Subgroup analyses of SII **(A)**, SIRI **(B)**, and PD.

To assess the consistency of the associations between the SII, SIRI, and PD across different patient profiles, we performed subgroup analyses based on predefined demographic and clinical characteristics (Figure 3). The MMSE used the following thresholds for cognitive impairment scores based on education: illiteracy, ≤17 points; 1–6 years, ≤19 points; and ≥7 years, ≤24 points (31). MoCA score of ≤26 points indicates cognitive impairment (32). The positive associations remained significant and consistent across all subgroups, including those stratified by age (<65 years vs. ≥65 years), sex (male vs. female), disease duration (<5 years vs. ≥5 years), disease stage (H&Y 1–2.5 vs. 3–5), and cognitive status (cognitively impaired vs. cognitively normal). Interaction tests confirmed no significant effect modification by any of these covariates (all P for interaction > 0.05). These findings demonstrate the robustness and broad generalizability of our conclusions across diverse population subgroups.

### ROC curves for the SII and SIRI in the diagnosis of PD

**Figure 4:**
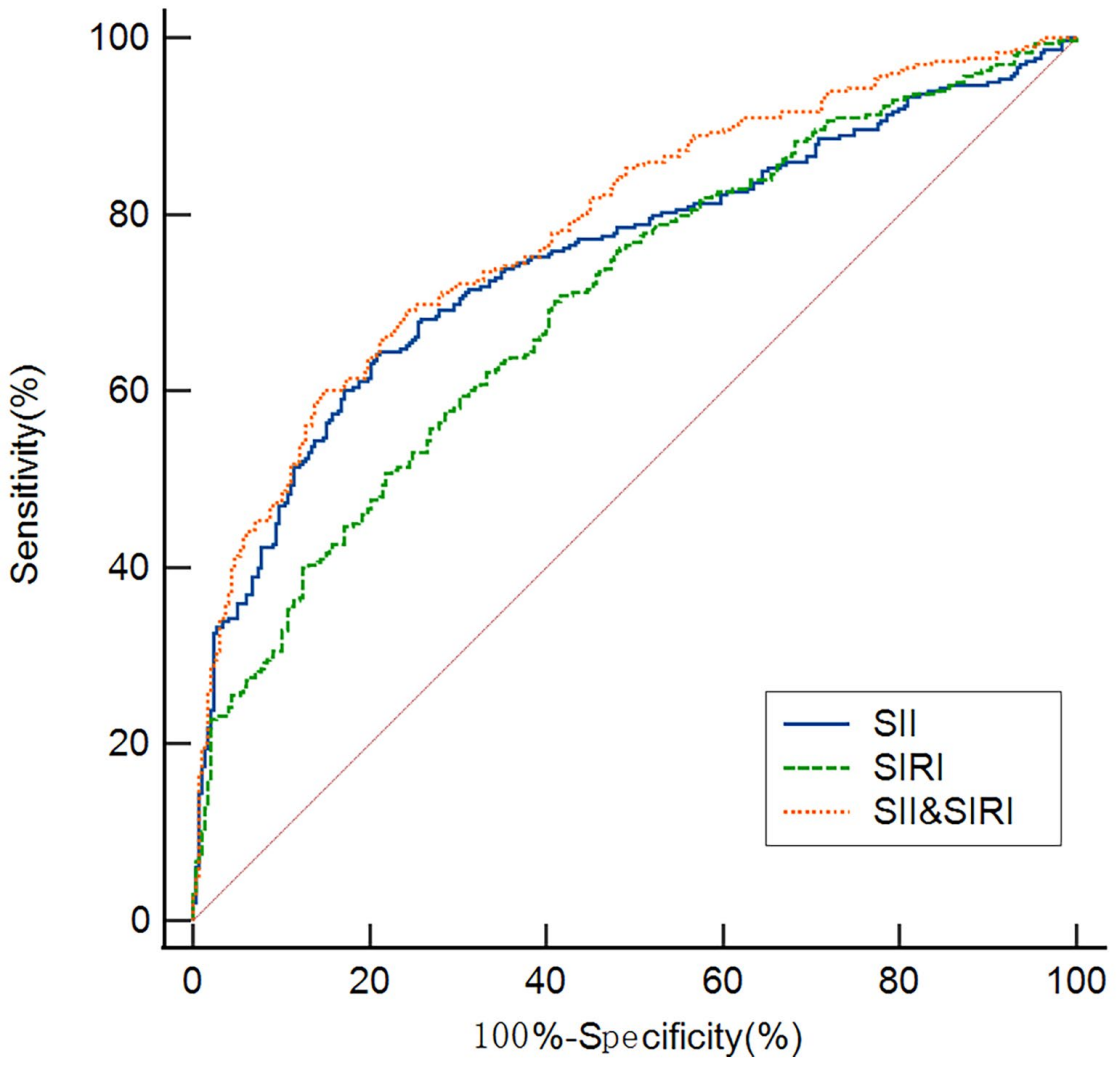
ROC curves demonstrating the discriminative ability of the SII, SIRI, and their combination in identifying patients with PD. The AUC were 0.750 for SII, 0.700 for SIRI, and 0.785 for the combined model.

As shown in Figure 4, ROC analysis was performed to evaluate the discriminative ability of SII and SIRI for PD. The SII achieved an AUC of 0.750 (95% CI: 0.722–0.793, *p* < 0.001), with an optimal cutoff of 416.23 yielding 64.45% sensitivity and 80.23% specificity. The SIRI showed an AUC of 0.700 (95% CI: 0.661–0.738, *p* < 0.001), with a cutoff of 0.73 corresponding to 7.91% sensitivity and 60.24% specificity. Notably, the combination of SII and SIRI improved diagnostic performance, yielding an AUC of 0.785 (95% CI: 0.757–0.825, *p* < 0.001) with 65.02% sensitivity and 83.17% specificity at a cutoff of 0.52.

## Discussion

Current diagnosis of PD primarily relies on core motor symptoms, yet the decades-long prodromal phase frequently leads to misdiagnosis in early stages (33). Compounding this challenge, substantial nigral dopaminergic neuron loss occurs before clinical manifestation (34), and advanced PD confers severe disability with significant socioeconomic burdens. These issues underscore the urgent need for reliable biomarkers enabling early detection. Growing evidence implicates inflammatory mechanisms in PD pathogenesis, prompting our investigation into the SII and SIRI as potential biomarkers. Our study yielded several key observations: PD patients demonstrated significantly elevated SII and SIRI levels compared to HCs, with both indices correlating with disease severity. Multivariable logistic regression confirmed their status as independent associated factors, while ROC analysis evidenced their discriminative capacity for PD identification. Furthermore, subgroup analyses revealed no significant interaction effects (all *p* > 0.05), indicating that the associations of the SII and SIRI with PD remained consistent across all predefined demographic and clinical strata. These results underscore the robustness and broad generalizability of our findings in diverse patient populations. Collectively, these findings establish elevated SII and SIRI as promising inflammatory biomarkers associated with PD.

The SII, integrating neutrophil, lymphocyte, and platelet counts, serves as a novel composite biomarker reflecting systemic inflammatory and immune status (35). Our study demonstrated significantly elevated SII levels in PD patients compared to HCs, with positive correlations between SII and UPDRS total and subscale scores, suggesting its potential utility in tracking PD progression-a finding consistent with prior reports (17). A binary logistic regression model demonstrated that the SII was an independent predictor of PD. Moreover, when the SII was employed as a categorical variable, after controlling for confounding variables, indicated that an elevated SII correlated with an increased likelihood of developing PD. While our results align with several previous studies (18, 19), the association between SII and PD remains controversial, as other groups have reported null findings (20, 21). Cohort characteristics may explain the discrepancies between the studies. For example, the work of Bissaco et al. (36) showed that SII is elevated in middle to advance disease compared to *de novo*. Therefore, future longitudinal studies should aim to recruit cohorts that stratify by important variables such disease stage and duration, motor subtype, cognitive impairment or neuropsychiatric comorbidities, sex, concomitant medication use, and co-existence of pro-inflammatory systemic conditions. Collectively, our work strengthens the evidence supporting SII as a clinically relevant inflammatory biomarker in PD.

The SIRI has emerged as an integrated inflammatory-immune biomarker with established prognostic value across multiple inflammatory conditions (22–24) and cardiovascular diseases (25–28). Although a recent study linked SIRI to anxiety symptoms in PD (29), its overall relationship with PD pathogenesis remains underexplored. Our study addresses this gap by demonstrating elevated SIRI levels in PD patients, correlations with clinical severity (UPDRS total and subscales), and independent predictive value for PD risk-assessed both as continuous and categorical variables. These findings broaden the potential role of SIRI in PD pathophysiology and highlight its possible utility in risk stratification. Nevertheless, given contradictory reports in the literature (20, 21), further validation through multicenter, large-scale studies is warranted to confirm these associations.

Our findings establish the elevated SII and SIRI are associated with the severity of PD and have potential utility as diagnostic biomarkers. However, the underlying pathophysiological mechanisms require further investigation. Several interconnected pathways may explain how elevated SII and SIRI contribute to PD pathogenesis. Firstly, neurovascular unit impairment represents a fundamental mechanism in PD progression. Evidence from clinical studies demonstrates increased BBB permeability in advanced PD stages (37, 38), facilitating the translocation of peripheral inflammatory mediators into the central nervous system. This pathological process is further supported by pharmacological evidence: non-steroidal anti-inflammatory drugs demonstrate potential neuroprotective effects through neutrophil count reduction (39). Our study demonstrated significantly elevated neutrophil counts in patients with PD compared to HCs, further supporting the involvement of neutrophils in PD pathogenesis. Secondly, the neuroimmune axis participates through coordinated cellular interactions. Th17-mediated immune responses orchestrate neutrophil recruitment to neural tissues, amplifying inflammatory cascades through degranulation processes (40). Central nervous system penetration of CD4^+^ and CD8^+^ T lymphocytes directly correlates with dopaminergic neurodegeneration (40, 41). Consistent with the findings of prior research (1, 42), we observed significantly lower plasma lymphocyte counts in patients with PD compared to HCs. While peripheral lymphopenia in PD patients may reflect compensatory trafficking of immune cells to cerebral compartments (43, 44). Thirdly, additional hematological components contribute to PD pathophysiology. Platelet-derived monoamine oxidase-B catalyzes the conversion of 1-methyl-4-phenyl-1,2,3,6 tetrahydropyridine (MPTP) to neurotoxic 1-Methyl-4-phenylpyridinium (MPP^+^) compounds (45), while *α*-synuclein exhibits monocyte-activating properties that correlate with disease severity (46). In conclusion, our results corroborate the positive correlation between systemic inflammation and PD risk. As composite inflammatory indices derived from routine hematological parameters, SII and SIRI offer practical value for risk stratification in PD. However, definitive mechanistic insights into their pathological roles warrant further multidisciplinary investigation.

Uric acid (UA) is widely recognized as a natural antioxidant that exerts neuroprotective effects through free radical scavenging and antioxidant activity in neurological disorders (47). A study of blood alterations during the PD prodromal phase revealed significantly lower serum UA levels in individuals at high risk of developing PD (48). Reduced serum UA concentrations were associated with increased PD incidence, accelerated disease progression, and exacerbated motor and cognitive impairments in patients receiving long-term dopaminergic therapy (49). Additional clinical observations have established correlations between decreased serum UA levels and poorer sleep quality in PD patients, suggesting its potential utility as a biomarker for sleep disturbances in this population (50). A previous study have also linked lower serum UA levels with motor function deterioration (51), while both serum UA and UA/creatinine ratio showed negative correlations with Hoehn-Yahr staging and served as independent negative marks of PD incidence and progression (52). In contrast to these findings, our univariate and multivariate analyses did not identify significant associations between serum UA levels and motor performance in PD, consistent with some previous reports (2, 17). We propose that discrepancies observed across studies may be explained by methodological differences, such as variations in the ethnic composition of study cohorts, limitations in sample size, heterogeneity in medication regimens, and differences in the distribution of disease severity.

Given the substantial clinical and pathological heterogeneity of PD, reliable risk stratification and progression monitoring cannot depend on any single biomarker. The integration of complementary biomarkers is therefore essential for enhancing predictive accuracy. In the present study, we employed ROC analysis to evaluate the combined diagnostic performance of the SII and SIRI. Our results demonstrate that both indices effectively discriminated PD patients from HCs, with SII exhibiting superior discriminatory power (AUC = 0.750) relative to SIRI (AUC = 0.700). The combination of SII and SIRI yielded an AUC of 0.785, representing a statistically significant improvement over either biomarker alone. However, the ROC curves show only modest discriminative ability (AUC < 0.80). This indicates that they are not suitable for use as independent diagnostic tools but may have value in combination with other clinical parameters or for identifying high-risk populations.

This study has several limitations that should be considered when interpreting the results: (1) As a cross-sectional study conducted exclusively in a Chinese population and cannot directly infer causality. Thus, future large-scale longitudinal studies involving diverse ethnic cohorts are required to validate and generalize these findings; (2) the absence of complementary inflammatory biomarkers limited our ability to correlate SII and SIRI with broader inflammatory profiles, thereby constraining a comprehensive understanding of neuroimmune interactions in PD; (3) although key clinical variables were adjusted for, unmeasured confounder-such as genetic predisposition, neuroimaging features, environmental exposures, and substance use history-may influence the observed associations; (4) we did not collect vaccination history. Vaccination can temporarily elevate platelet count and other inflammatory markers (e.g., cytokines) which could impact the study results (53); (5) the multivariable analyses are under-adjusted. Key clinical variables such as on disease duration and LEDD are not included. Their omission reduces the strength of the claims regarding “independent” associations; and (6) while SII and SIRI reflect systemic inflammation, they lack specificity to neuroinflammatory pathways and can be affected by non-neurological conditions, complicating interpretation of their direct roles in PD pathogenesis.

## Conclusion

In summary, our study demonstrates that elevated SII and SIRI are significantly associated with PD severity and represent promising composite biomarkers for clinical assessment. Importantly, the combination of these indices shows superior predictive value compared to individual markers, suggesting their potential utility in multidimensional evaluation approaches. Nevertheless, further validation through prospective multicenter studies and deeper investigation into their pathophysiological mechanisms are warranted to establish their definitive role in PD management.

## Data Availability

The raw data supporting the conclusions of this article will be made available by the authors, without undue reservation.
